# Neurasthenia and Its Treatment

**Published:** 1874-05

**Authors:** Geo. M. Beard

**Affiliations:** New York


### Neurasthenia and its Treatment.

New York, March 23, 1874.

Messrs. Editors.—In Dr. Fisher's article on Neuriasis, recently published in your Journal, I am credited with having introduced the term "neurasthenia" to the profession. This claim, I have recently learned, is not strictly just. In Dunglison's Medical Dictionary (1860) I find the following:—"Neurastheni, *Neurodynamia Debilitas Nervosa.* Debility or impaired activity of the nerves; from *νευρον*, 'a nerve,' and *ασθενεια*, 'debility.'"

Dr. Van Heusen, in a paper published several years ago, spoke of neurasthenia as an old term; but I supposed that he was in error. It seems, therefore, that the word neurasthenia has at some time been in use, and it is also clear that it had been pretty generally forgotten. There is, so far as I can learn, no evidence that it had ever been used to represent any very definite state, or that a disease worthy of that name had ever been recognized. I devised the term independently, from νευρον, "a nerve," α privative, and σθενεια, "strength," and tried to make a differential diagnosis between neurasthenia and anæmia, with which it is usually confounded. Dr. Austin Flint had previously written a short chapter on nervous asthenia. I did not intend to include under the term neurasthenia all distinctly hysterical symptoms, but rather the vast array of phenomena that appear in the stages of nervous exhaustion, through which hysterical patients pass before they develope those emotional disturbances that are generally regarded as diagnostic of hysteria. As I understand Dr. Radcliffe, neuriasis covers about the same ground. Neurasthenic patients do not of necessity ever develope the extreme symptoms of hysteria, and, conversely, those patients who are affected with what I call mental hysteria, which depends exclusively on the *mental* organization—the predominance of emotion over reason—do not of necessity ever pass through the stage of neurasthenia.

It is not my desire here to write an essay on the subject, but I may, perhaps, briefly suggest the method of treatment that I have found of very great efficacy in neurasthenia, namely, central galvanization and the use of cod-liver-oil emulsion. My method of central galvanization has already been described in this Journal. The cod-liver-oil emulsion has also been published in the *Archives of Scientific and Practical Medicine;* but it is not yet fully appreciated. The prescription of a cod-liver-oil emulson was originally given me by Dr. J. B. Andrews, of the Utica Insane Asylum. I have modified this perscription in various ways, and have experimented with it quite largely, and have recommended it to many of my professional friends. It takes a long time to make it, and the majority of druggists will slight it unless they are assured it will be ordered in large quantities. The latest modification that I employ is the following. If desired, Fowler's solution may be added to it. One of my pa ients, a physician, has added strychnine to it.

℞. Glyconin, ℨix.;

Ol. morrhuæ, ℥iv.;

Spts. ammon, arom., ℨi.;

Vini xerici, ℥ii.;

Acid. phos. dil., ℥ss.;

Ol. amygdal, amar., gtt. ij.; dissolved in alcohol, ℨij. M.

Put the glyconin first in the mortar, then add the oil by drops, stirring briskly all the time. When this process is completed, you will have a mass looking like, and having the consistency of, soft butter. Then add the other ingredients in the order mentioned; add them slowly, stirring all the time. The glyconin is made by beating the yolks of eggs with a spatula until they are well broken, then add an equal measure of glycerine. It requires one or two hours to make it.

The above preparation, when *properly prepared*, robs cod-liver-oil of its terrors. The taste of the oil is pretty nearly destroyed, and no one, however fastidious, objects to it. When properly prepared, it will keep for months, if not years. Mr. Close, a pharmacist of Brooklyn, who introduced the glyconin modification in the emulsion, lets the oil drop out of a small bottle through a glass tube, and thus avoids the danger of pouring it in too fast. This emulson, when made according to the above directions, with sufficient care, time and labor, is without question the best emulsion of the kind now before the profession. The original prescription, of which the above is a modification, is, or was, used in the Utica Insane Asylum in large quantities, and with satisfactory results. One druggist, to whom I gave the prescription, makes several gallons at a time and keeps it always on hand. Consumptives, who must take cod-liver-oil, and yet cannot bear it, find this emulsion agreeable, and as far as I can learn, as useful as the oil when taken alone.

My own experience with the emulsion has been confined mostly to its use in hysteria, neurasthenia and allied affections, and I know of no single prescription that does so much good and so little harm in all these aneuric disturbances. I earnestly recom- it to the profession, and especially to those who are giving special attention to diseases of the nervous system.

The dose ranges between a dessertspoonful and two tablespoonfuls. If it tastes badly and keeps badly, the fault is in the druggist. Yours, &c. GEO. M. BEARD.

—*Correspondence Boston Med. Journal.*

